# Analysis of rare thalassemia genetic variants based on third-generation sequencing

**DOI:** 10.1038/s41598-022-14038-8

**Published:** 2022-06-14

**Authors:** Cuiting Peng, Haixia Zhang, Jun Ren, Han Chen, Ze Du, Tong Zhao, Aiping Mao, Ruofan Xu, Yulin Lu, He Wang, Xinlian Chen, Shanling Liu

**Affiliations:** 1 Center of Prenatal Diagnosis, Department of Medical Genetics, West China Second University Hospital, Sichuan University, Chengdu, China; 2 Key Laboratory of Birth Defects and Related Diseases of Women and Children, Sichuan University, Ministry of Education, Chengdu, China; 3 Berry Genomics Corporation, Beijing, 102200 China; 4 Department of Obstetrics and Gynecology, West China Second University Hospital, Sichuan University, 17 South Renmin Road, Chengdu, China

**Keywords:** Genetics, Medical genetics, Diseases, Anaemia

## Abstract

Thalassemia is a group of common hereditary anemias that cause significant morbidity and mortality worldwide. However, precisely diagnosing thalassemia, especially rare thalassemia variants, is still challenging. Long-range PCR and long-molecule sequencing on the PacBio Sequel II platform utilized in this study could cover the entire *HBA1*, *HBA2* and *HBB* genes, enabling the diagnosis of most of the common and rare types of thalassemia variants. In this study, 100 cases of suspected thalassemia were subjected to traditional thalassemia testing and third-generation sequencing for thalassemia genetic diagnosis. Compared with traditional diagnostic methods, an additional 10 cases of rare clinically significant variants, including 3 cases of structure variants and 7 cases of single nucleotide variations (SNVs) were identified, of which a case with − α^3.7^ subtype III (− α^3.7III^) was first identified and validated in the Chinese population. Other rare variants of 11.1 kb deletions (− 11.1/αα), triplicate α-globin genes (aaa^3.7^/αα) and rare SNVs have also been thoroughly detected. The results showed that rare thalassemia variants are not rare but have been misdiagnosed by conventional methods. The results further validated third-generation sequencing as a promising method for rare thalassemia genetic testing.

## Introduction

Thalassemia is a group of common hereditary anemias that cause significant morbidity and mortality worldwide, especially in Southeast Asian, Middle Eastern and Mediterranean populations^1,2^. α-Thalassemia (α-thal) and β-thalassemia (β-thal) are two main types of thalassemia caused by mutations in the *HBA1/2* and *HBB* genes respectively, which result in abnormal α- and β-globin synthesis and defective hemoglobin structure^3,4^.

The clinical manifestations of thalassemia vary greatly and are usually directly related to the degree of globin chain reduction. There are three types of thalassemia carrier states (silent, minor and intermedia) and one disease state (major) according to the amount of globin chain and the disease severity^5,6^. α-Thalassemia major is also known as Hb Bart's hydrops fetalis, and affected fetuses usually die in utero or shortly after birth due to severe anemia and lack of oxygen^7^. Whereas, affected individuals with the most severe form of β-thalassemia usually can survive, but only with regular blood transfusions and iron chelation therapy^8,9^. It has been estimated that approximately 5% and 1.5% of the population worldwide are carriers of α- and β-thalassemia genetic mutations. Thus, a large number of children are still born annually with hemoglobin disorders, which cause severe birth defects and place heavy burdens on society and families^3,10^.

Due to the complexity of thalassemia genetics and genotype–phenotype correlation, precisely diagnosing thalassemia patients and carrier status are still challenging^11^. Effective and accurate molecular diagnosis methods are urgently needed to identify rare clinically significant variants of thalassemia genes. For traditional thalassemia genetic diagnosis, the reverse dot blot hybridization, Sanger sequencing, GAP-PCR and Multiple ligation-dependent probe amplification (MLPA) can be utilized to detect the prevalent variants, including SNVs, indels and copy number variations in *HBB* and *HBA1/2*^12^. More recently, next-generation sequencing (NGS) based on PCR for targeted exons and selected intronic regions has shown advantages in thalassemia gene screening^13^. However, for rare variants not located in regular regions or variants in homologous regions of *HBA1* and *HBA2*, conventional methods and short-read based NGS methods may lead to missed diagnosis or even misdiagnosis. Currently, a methodology based on third-generation sequencing (TGS) named Comprehensive Analysis of ThalaSsaemia Alleles (CATSA) was developed and validated for comprehensive thalassemia screening^14,15^. Based on long-range PCR and long-molecule sequencing on the PacBio Sequel II platform, the CATSA method is adequate to cover the entire gene region and enable the diagnosis of common and rare types of thalassemia variants. To further investigate the potential of long-molecule sequencing in rare thalassemia carrier testing, we enrolled 100 cases that either showed an abnormal hematology phenotype or hemoglobin electrophoresis, but had negative conventional genetic diagnosis results. In this study, the CATSA method detected an extra 10 cases of clinically significant variants from *HBA1/2* and *HBB*, including 3 cases of SVs and 7 cases of SNVs, of which a very rare subtype of − α^3.7^ named − α^3.7^ subtype III (− α^3.7III^) was first identified in the Chinese population and validated by Sanger sequencing. Other rare genotypes with 11.1 kb deletions (− 11.1/αα) or triplicate α-globin genes (aaa^3.7^/αα) and rare SNVs can be thoroughly detected in one test. The study further validated long-molecule sequencing based CATSA as an efficient and valuable method in the diagnosis of rare thalassemia.

## Patients and methods

### Patients

A total of 100 patients highly suspected of having thalassemia gene mutations were included in this study. Informed consents were obtained from all participants or from a parent and/or legal guardian if participants are under 18. The inclusion criteria for the enrolled patients were: either (1) routine hematology examinations showed abnormal hemoglobin of HbA2 < 2.3% or HbA2 ≥ 3.2% or elevated HbF; or (2) the blood test showed abnormalities with mean corpuscular volume (MCV) ≤ 80 fL for adults and/or mean corpuscular hemoglobin (MCH) ≤ 27 pg; or (3) the genotype identified by conventional genetic testing could not adequately explain their clinical phenotype. The age range for the included 18 children was 25 days to 2.5 years and the mean age for the other 82 adults was 30.5.

### GAP-PCR testing for large-deletion α-thalassemias

Single-tube multiplex Gap-PCR was performed for the three common α-thalassemia deletions including − ^SEA^ (Southeast Asia), − α^3.7^ (rightward) and − α^4.2^ (leftward) according to the manufactorer’s protocol. (Yaneng Bioscience, Shenzhen, China).

### Reverse dot blot hybridization assays

A PCR reverse dot-blot (PCR-RDB) assay was performed for three common non-deletional α-thalassemia mutations including Hb Constant Spring (Hb CS, HBA2: c.427 T > C), Hb Quong Sze (Hb QS, HBA2: c.377 T > C), and Hb Westmead (Hb WS, HBA2: c.369C > G), and 17 β-thalassemia mutations including − 32 (C > A) (HBB: c. − 82C > A), − 30 (T > C) (HBB: c. − 80 T > C), -29 (A > G) (HBB: c. − 79A > G), − 28 (A > G) (HBB: c. − 76A > G), CAP + 40–43 (− AAAC) (HBB: c. − 11_ − 8delAAAC), initiation codon (T > G) (HBB:c.2 T > G), codons 14–15 (+ G) (HBB: c.45_46insG), codon 17 (A > T) (HBB: c.52A > T), codon 26 (or Hb E) (G > A) (HBB: c.79G > A), codons 27/28 (+ C) (HBB: c.84_85insC), codon 31 (− C) (HBB: c.94delC), codons 41–42 (–TTCT) (HBB: c.126_129delCTTT), codon 43 (G > T) (HBB: c.130G > T), codons 71–72 (+ A) (HBB: c. 216_217insA), IVS-I-1 (G > T) (HBB: c.92 + 1G > T), IVS-I-5 (G > C) (HBB: c.92 + 5G > C), and IVS-II-654 (C > T) (HBB: c.316-197C > T). (Yaneng Bioscience, Shenzhen, China).

### Long PCR based third-generation sequencing and data analysis

Experiments were performed as previously described^14^. Briefly, genomic DNA was amplified by PCR with primers targeting the majority of known structural variations, SNVs and indels in the *HBA1*, *HBA2* and *HBB* genes. Barcoded adaptors were ligated to the PCR products to construct individual sequencing libraries. Then, each library was quantified and pooled together by equal mass. After purification and quantification, the pooled library was converted to a SMRTbell library with Sequel Binding and Internal Ctrl Kit 3.0 (Pacific Biosciences) and sequenced on the Sequel II platform (Pacific Biosciences) under CCS mode. Then raw subreads were analyzed by CCS software (Pacific Biosciences) to generate CCS reads, debarcoded by lima in the Pbbioconda package (Pacific Biosciences) and aligned to genome build hg38 by pbmn2 (Pacific Biosciences). Finally, structural variations were identified according to the HbVar, Ithanet and LOVD databases. SNVs and indels were identified by FreeBayes1.3.4 (https://www.geneious.com/plugins/freebayes; Biomatters, Inc., San Diego, CA).

### Variant confirmation

All the SNVs detected by third-generation sequencing were further confirmed by Sanger sequencing using specific primers. The deletion variants and α-globin gene triplication variants were confirmed by specific PCR assays and agarose electrophoresis according to the manufacturer’s protocol (Yaneng Bioscience, Shenzhen, China).

### Ethical approval

The present study was approved by the Ethics Committee of West China Second Hospital of Sichuan University. All methods were carried out in accordance with guidelines and regulations from the Declaration of Helsinki.

## Results

### Detection and identification of rare clinically significant SNVs

**Figure 1:**
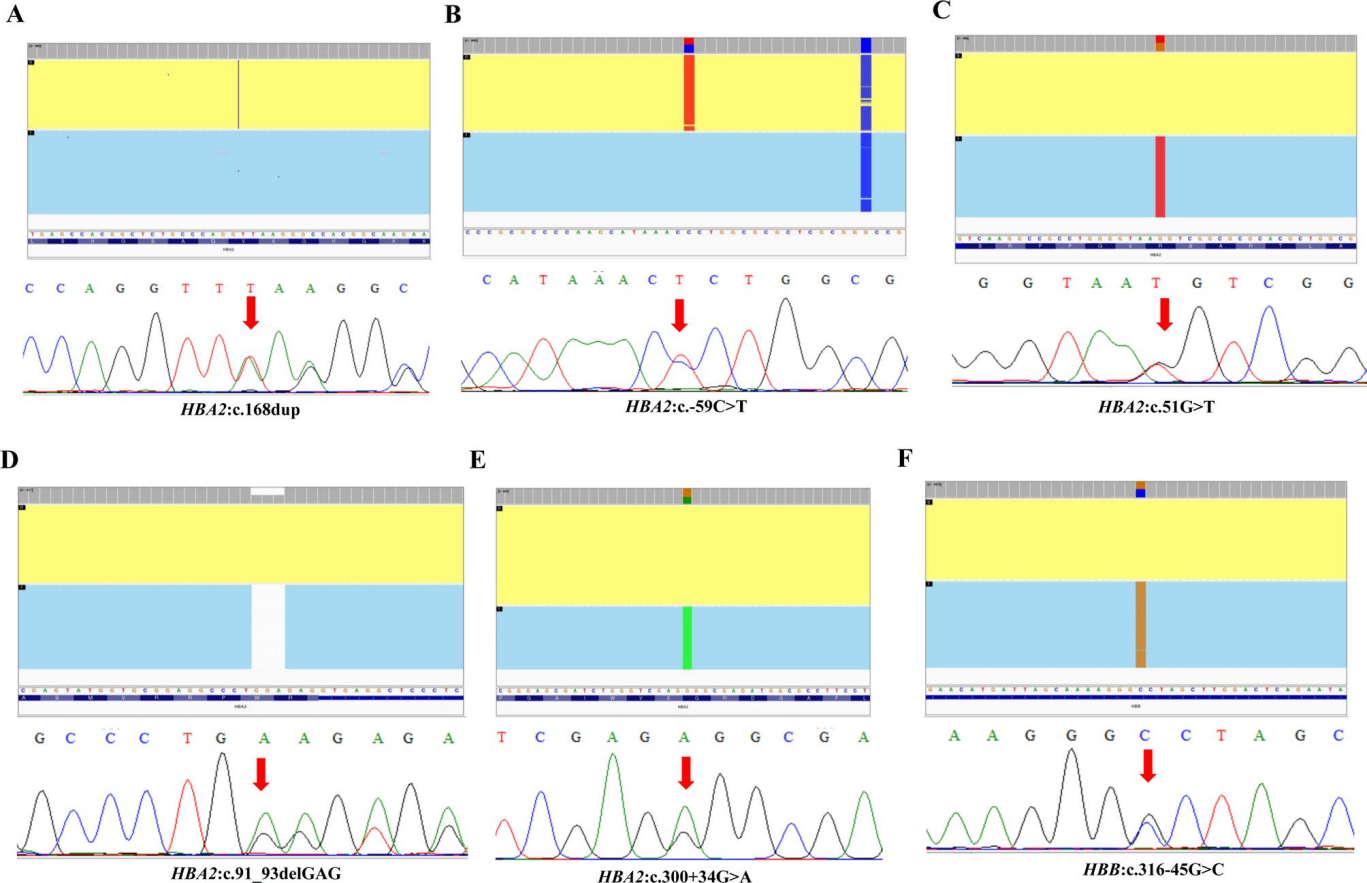
Identification and verification of clinically significant SNVs. (**A**–**F**) The six SNVs identified in the *HBA2* and *HBB* genes by third-generation sequencing. The top graphs are the results exported from Integrative Genomics Viewer (IGV), and the bottom graphs are the Sanger sequencing results for each variant.

In this study, we identified 7 cases of rare SNVs that can be ranked as clinically significant variants including *HBA2* SNVs (c.168dup, c. − 59C > T, c.51G > T, c.91_93delGAG, and c.300 + 34G > A) and *HBB* c.316–45G > C (Table 1). All the long molecular sequencing data were verified by specific PCR and Sanger sequencing (Fig. 1). Among them, heterozygous *HBA2*:c.168dup, also known as codons 55/56 (+ T) could cause α^+^-thalassemia due to a frameshift mutation of the α2-globin gene, which could better explain the phenotype of participant #2 with mild microcytosis and hypochromia (Table 2)^16^. Variant *HBA2*:c. − 59C > T (participant #3) is one type of point mutation found in the promoter of the α2-globin gene that affects *HBA2* transcription and protein synthesis^17^. Additionally, heterozygosity of *HBA2*:c.51G > T (CD16 (AAG > AAC or AAT)) and *HBA2*:c.91_93delGAG (codon 30(–GAG)) may influence protein translation, thus resulting in the abnormal hemoglobin and hematology phenotypes for participants #4 and #5 (Table 2)^18,19^.

**Table 1:** Single nucleotide variations (SNVs) and Structural variation (SV) regions in HBA2 and HBB genes detected by CASTA.

Participant number	Gap-PCR and reverse dot blot hybridization assays results		CATSA results		Variant verification
	α thalassemia	β thalassemia	α thalassemia	β thalassemia	
1	− SEA/αα	N	− SEA/αα HBA2: c.*82G > A HBA2: c. + 92A > G HBA2: c.*98 T > C	N	Electrophoresis
2	αα/αα	N	HBA2: c.168dup	N	Sanger sequencing
3	αα/αα	N	HBA2: c. − 59C > T	N	Sanger sequencing
4	αα/αα	N	HBA2: c.51G > T	N	Sanger sequencing
5	αα/αα	N	HBA2: c.91_93delGAG	N	Sanger sequencing
6	αα/αα	N	HBA2: c.300 + 34G > A	N	Sanger sequencing
7	αα/αα	N	N	HBB: c.316 − 45G > C	Sanger sequencing
8	αα/αα	N	− α3.7/αα	N	Sanger sequencing
9	αα/αα	N	− 11.1/αα	N	–
10	αα/αα	N	ααα^3.7^/αα	N	Electrophoresis

**Table 2:** Hematology examination and hemoglobin electrophoresis results in patients with detected clinically significant variants.

Participant number	Genotype	Age (y)	Hb (g/L)	MCV (fL)*	MCH (pg)	HbA2 (%)	HbF (%)
1	− SEA/αα HBA2: c.*82G > A HBA2: c. + 92A > G HBA2: c.*98 T > C	2	95	55.8	15.8	0.8	0.4
2	HBA2: c.168dup	28	99	79.9	25.8		
3	HBA2: c. − 59C > T	28	99	74.9	23.6	2.5	0.0
4	HBA2: c.51G > T	32	135	93.4	31.9	1.8	0.7
5	HBA2: c.91_93delGAG	2	118	70.1	23.1		
6	HBA2: c.300 + 34G > A	30	135	87.4	30.3	1.6	16.3
7	HBB: c.316 − 45G > C	29	97	78.4	22.6		
8	− α3.7/αα	31	114	80.3	25.0	3.9	0.0
9	− 11.1/αα	37	114	82.6	25.9	2.3	0.0
10	ααα^3.7^/αα	30		91.5	30.0	3.3	

*The reference value for MCV is age-related especially for the age of under 2. Thus, for case 1 and case 5 who are 2 years-old, the reference MCV are 70.3–87.9 according to the standard of our hospital.

### Multiple mutations in the polyadenylation signal site and compound − ^SEA^/αα

**Figure 2:**
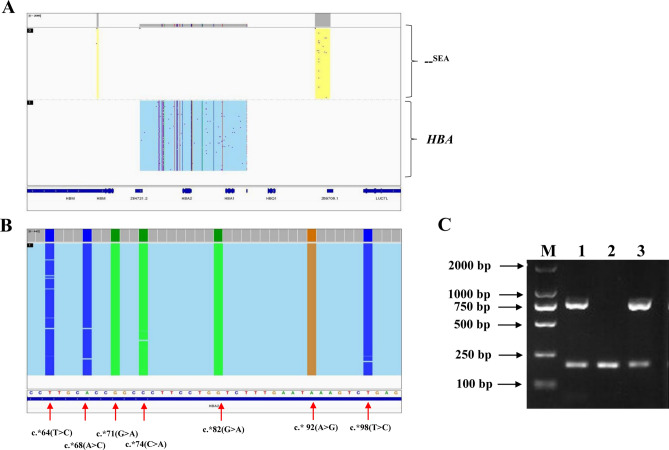
Identification of multiple mutations in the polyadenylation signal site and compound − ^SEA^/αα. (**A**) − ^SEA^/αα identified by third-generation sequencing. (**B**) Seven mutations in the polyadenylation signal site identified by third-generation sequencing. (**C**) Identification by specific PCR and agarose gel electrophoresis. M: maker; 1: positive control for the HBA2 fusion gene; 2: negative control; 3: sample for participant #1. The original gels are presented in Supplementary Fig. 1.

Participant #1 showed a Hb H disease-like abnormal hematology phenotype of significantly decreased MCV and MCH, together with abnormal hemoglobin (0.8% of HbA2). Through screening of the common thalassemia deletions or mutations, only heterozygous Southeast Asian deletion (− ^SEA^/αα) was identified, which might not be able to explain the abnormal Hb electrophoresis. In this study, the third-generation sequencing further identified multiple mutations at the polyadenylation signal site in the α2-globin gene, including *HBA2*:c.*64(T > C), c.*68(A > C), c.*71(G > A), c.*74(C > A), c.*82(G > A), c.* 92(A > G) and c.*98(T > C) (Table 1, Fig. 2). The results were further verified by specific PCR and agarose gel electrophoresis (Yaneng Bioscience) (Fig. 2C). These multiple mutations in the polyadenylation signal site in combination with the − ^SEA^/αα genotype may lead to the phenotype of Hb H disease^20,21^.

### Identification of − α^3.7^ subtype III

**Figure 3:**
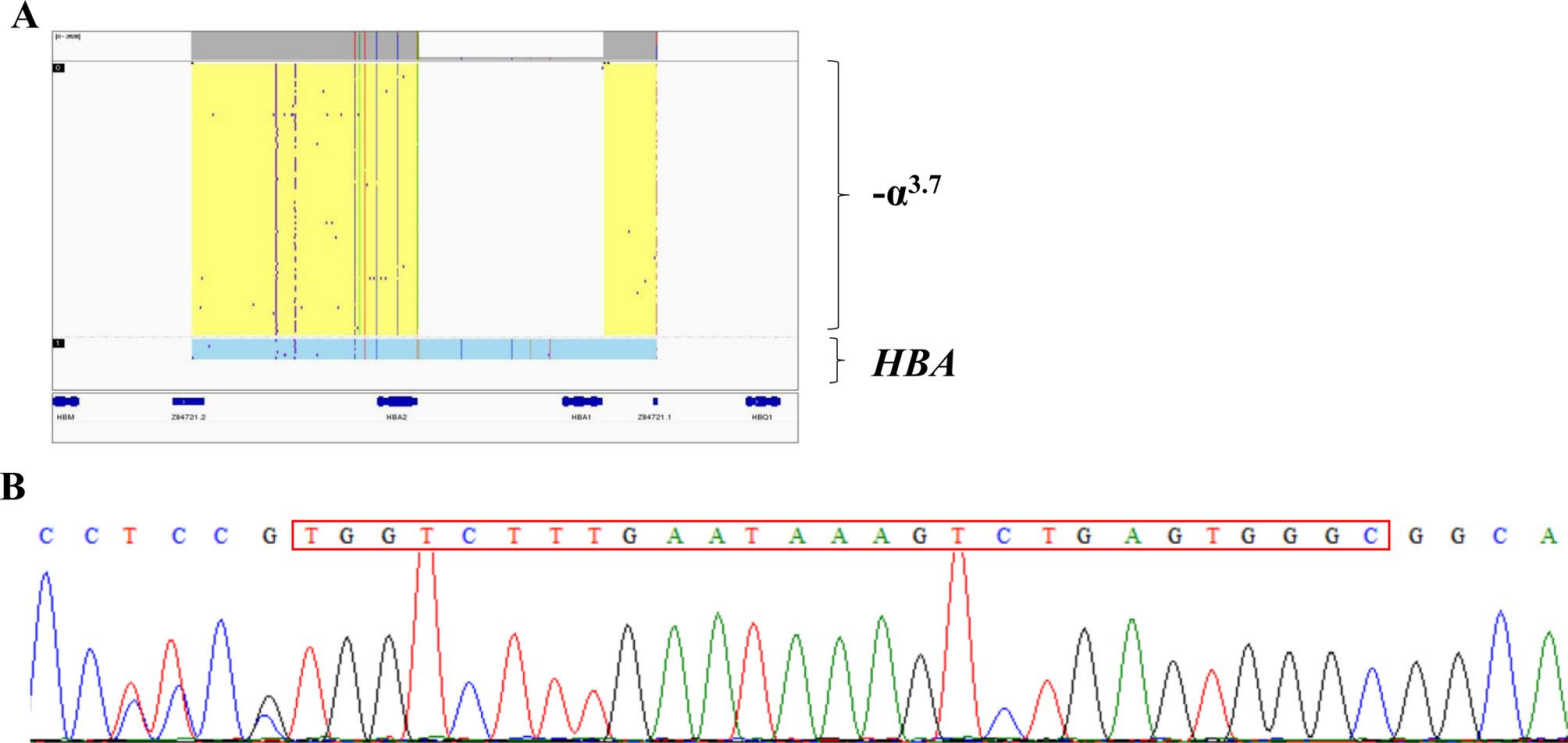
Identification and verification of -α^3.7^ subtype III. (**A**) The 3.8 kb deletion identified by third-generation sequencing. (**B**) Verification by Sanger sequencing. The sequences in the red box are these homologous sequences shared by *HBA1* (chr16:173,680–173,706) and *HBA2* (chr16:177,492–177,518).

Through third-generation sequencing, we identified a rare deletion of 3.8 kb involving *HBA2* and *HBA1* (chr16:173,707–177,518 based on sequencing analysis) in participant #8. The exact deletion breakpoints were also inferred by Sanger sequencing (Table 1, Fig. 3). Since the short segment of ‘TGGTCTTTGAATAAAGTCTGAGTGGGC’ could be located at chr16:173,680–173,706 and chr16:177,492–177,518 based on sequence alignment, the breakpoint should be located at chr16:173,707 and chr16:177,518. The corresponding HGVS nomenclature for this type of deletion was NG_000006.1: g.34570_38382del 3812 bp, which is a very rare type of − α^3.7^, that is − α^3.7^ subtype III^22^. To our knowledge, this is the first reported case of the − α^3.7^ subtype III in the Chinese population. The results also verified third-generation sequencing as a valuable method for rare thalassemia diagnosis.

### Identification of a rare deletion of 11.1 kb in *HBA1/2*

**Figure 4:**
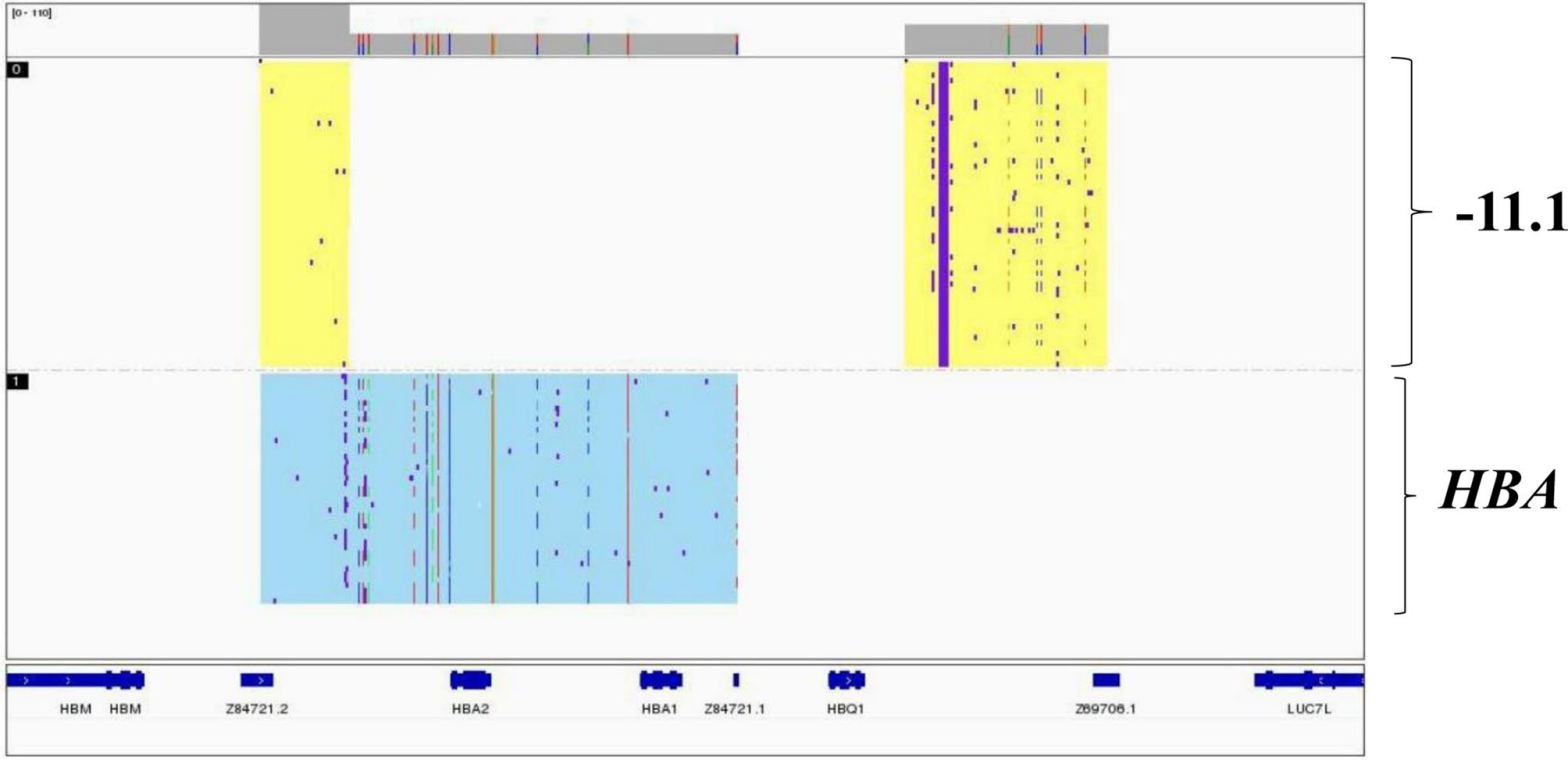
Identification of a rare deletion of 11.1 kb in *HBA1/2*. The 11.1 kb deletion identified by third-generation sequencing.

Another rare deletion of 11.1 kb involving the *HBA2* and *HBA1* genes was identified in participant #9, which could not be easily identified by conventional methods (Table 1, Fig. 4). This deletion, which has the HGVS nomenclature of NC_000016.9:g.(220831_220860)_(231920_232003)del, resulted in a type of α^0^-thalassemia that has been previously reported in the Chinese population^19^. The carriers with this deletion usually show mild α-thalassemia with hypochromic microcytic phenotypes (Table 2).

### Detection of an α-globin gene triplication

**Figure 5:**
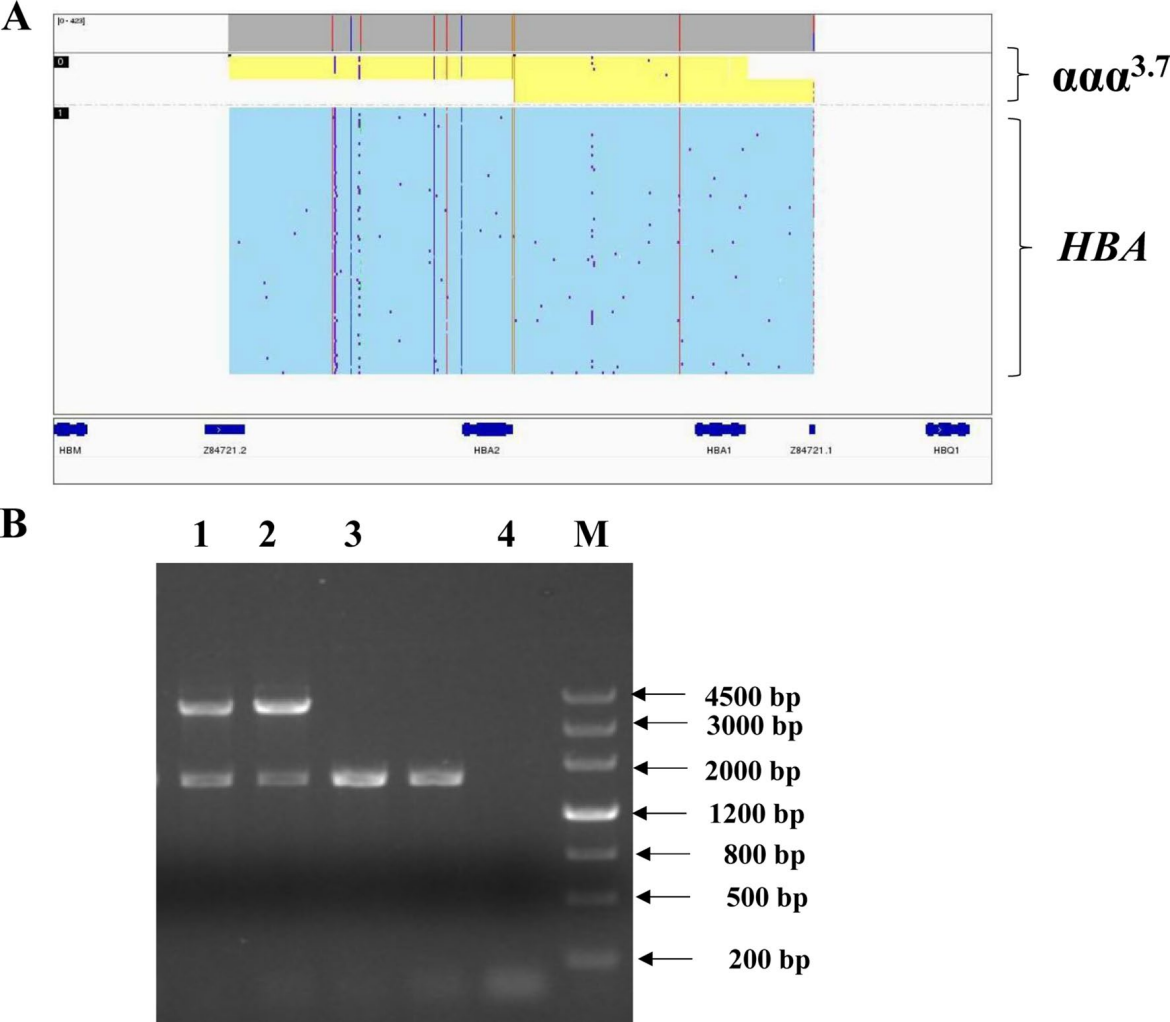
Identification and verification of α-globin gene triplication. (**A**) α-Globin gene triplication of ααα^3.7^/αα identified by third-generation sequencing. (**B**) Identification by specific PCR and agarose gel electrophoresis. M: Maker; 1:sample for participant #10; 2: positive control for ααα^3.7^/αα; 3: negative control; 4: blank. The original gels are presented in Supplementary Fig. 2.

One case of α-Globin gene triplication (ααα^3.7^/αα) was found in this study (participant #10). The results were further validated by specific PCR and agarose electrophoresis (Yaneng Bioscience) (Table 1, Fig. 5). Carriers with this α-globin gene triplication usually show normal results for routine hematology examination but abnormal HbA2 content^23^. In addition, if compounded with β-thalassemia, patients usually show intermediate β-thalassemia phenotypes.

## Discussion

Conventional genetic testing methods including gap-PCR, RDB, MLPA and Sanger sequencing can only detect common variants such as − α^3.7^, − α^4.2^, − ^SEA^, − α^27.6^, − α^21.9^ or other known SVs and SNVs, which are very limited. Since more than 2000 thalassemia or abnormal hemoglobin-related variant sites have been reported, it is necessary to develop new methods to implement in thalassemia diagnosis, especially for these rare variants. Recently, third-generation sequencing technology also known as long-molecule sequencing, has emerged as an incomparable method in genetic diagnosis with many advantages, including long reads, high accuracy, single molecule resolution and no GC preference^24^. The long reads could cover many rare gene loci, and their PCR-free characteristic made it possible to reflect the real arrangement in the genome. The method designed for thalassemia carrier screening based on third-generation sequencing on the Pacific Biosciences Sequel II platform has been optimized in recent years to detect thalassemia genes with high efficiency and accurate, also with acceptable time and cost.

Through third-generation sequencing for full-length of *HBA1*, *HBA2* and *HBB* genes, multiple variants may be recognized in one sample. Thus, it is crucial to evaluate the correlation between genotype and phenotype to further identify clinically significant variants^25^. The SNVs reported in this study, including *HBA2* SNVs (c.168dup, c. − 59C > T, c.51G > T, c.91_93delGAG, and c.300 + 34G > A) and *HBB* c.316–45G > C, all showed some evidence related to abnormal hematology phenotypes and hemoglobin results. Carriers usually have a normal phenotype, but tend to develop intermediate thalassemia when compounded with other variant types. Most of these participants are at the appropriate age and are willing to procreate. Thus, our results could pave the way for further prenatal diagnosis or even preimplantation genetic testing (PGT) to prevent the incidence of thalassemia^26^.

**Figure 6:**
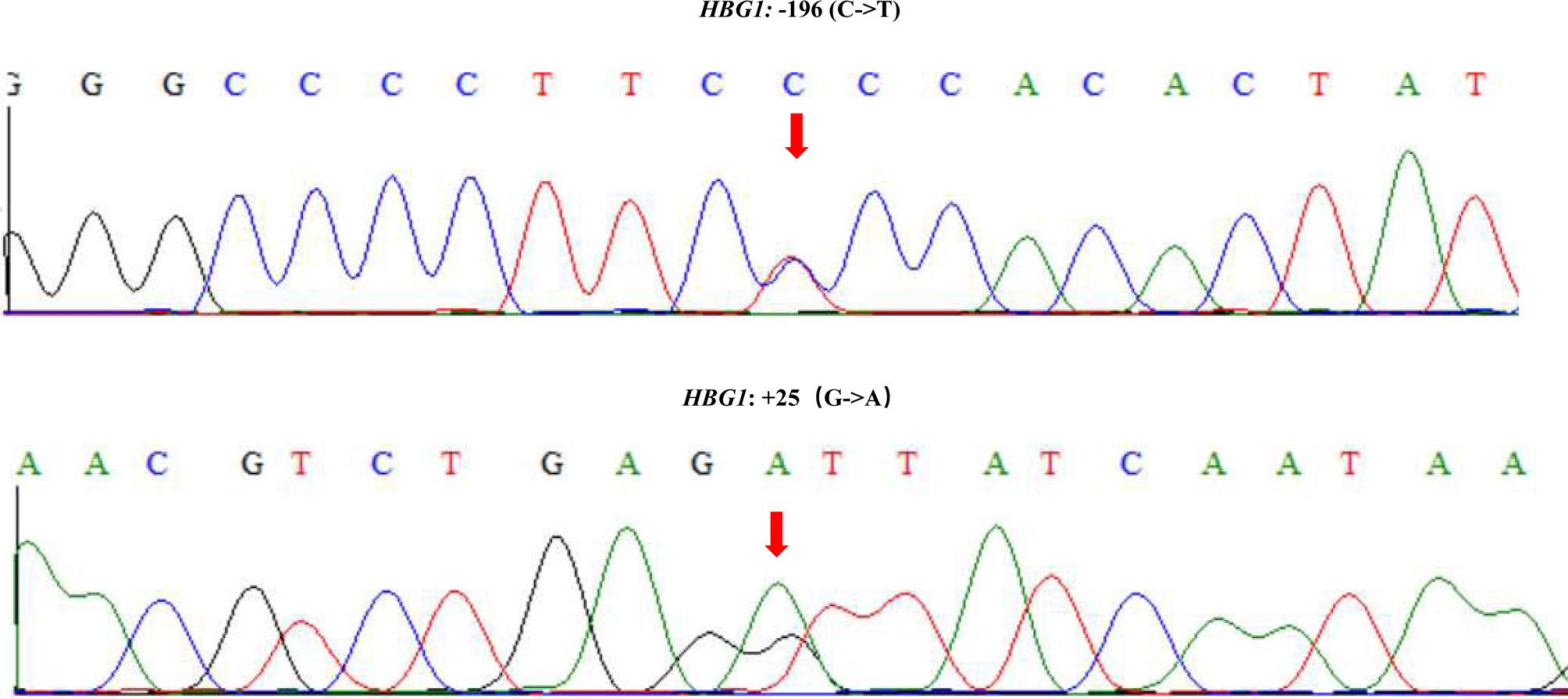
Identification of SNVs in HBG1 by Sanger sequencing. The two SNVs of *HBG1*: − 196 (C − > T) (top) and *HBG1*: + 25 (G − > A) (bottom) identified by Sanger sequencing for the full-length of *HBG1* gene.

It is worth mentioning that participant #6 showed an increased HbF content of 16.3%, yet only one clinically significant variant of *HBA2* c.300 + 34G > A was identified. We suspected that some other HPFH-causing variants in the gamma globin genes *HBG1* or *HBG2* may be responsible for the increased HbF level^27^. Thus, we conducted full-length Sanger sequencing for *HBG1* and *HBG2* genes. Two variants of − 196 (C − > T) and + 25 (G − > A) in the promoter region of *HBG1* were found (Fig. 6). Among these genes, − 196 (C − > T), also named *HBG1*:c. − 249C > T, may be responsible for the high expression of the gamma-globin gene, thus causing the increased HbF level according to previous studies^28,29^. The results suggested that it may be necessary to design more primers to detect other thalassemia-related genes, such as *HBG1* and *HBG2,* in the future, to implement comprehensive screening.

Moreover, we found one case with multiple mutations in the polyadenylation signal site compound with − ^SEA^/αα, which showed a rare Hb H disease. These multiple mutations led to a fusion between the α2 and ψα1 genes, which may affect mRNA transcription and termination. Thus, α2-globin gene polyA mutation in combination with − ^SEA^/αα may lead to Hb H disease. This patient is a 2-year-old boy with significantly decreased MCV and MCH, together with abnormal hemoglobin. Through conventional thalassemia genetic screening, only a heterozygous − ^SEA^/αα was found which is not consistent with his phenotype. Thus, third-generation sequencing provided a more efficient method for rare thalassemia gene testing for this case. Based on its hematological phenotype and clinical manifestations for Hb H disease, it will be necessary for the patient to seek further family analysis and genetic counselling.

Most importantly, we reported a case with a 3.8 kb deletion in *HBA2* that shared almost the same deletion junctions with the rare − α^3.7^ subtype III, which was the first reported case of the − α^3.7^ subtype III in the Chinese population. There are at least three subtypes of − α^3.7^ in the population based on the different recombination sites, that is − α^3.7^ I, − α^3.7^ II and -α^3.7^ III. The prevalence and population distribution of each subtype are different, among which − α^3.7^ subtype III is extremely rare in the population compared with the other two subtypes. Although relatively rare, the − α^3.7^ subtype III has been noted with high frequency in people in Pacific Island nations such as Micronesia and Polynesia and was also found with approximately 2% of α^+^-thalassemia alleles in the southern Thai population^30^. In our study, − α^3.7^III was discovered for the first time in the Chinese population using third-generation sequencing. The − α^3.7^ subtype III is prone to be undiagnosed since most of the conventional detection methods are designed for the identification of the other two subtypes. The PCR primers used in those PCR kits are usually located around the breakpoints of − α^3.7^ subtype III and homologous sequences in *HBA1* could also lead to miss diagnoses. The long reads of third-generation sequencing could better cover the whole sequences of *HBA1* and *HBA2*, thus most of the rare deletions or duplications in those two genes can be thoroughly identified.

## Conclusions

In conclusion, in this study we identified 10 rare clinically significant variations through third-generation sequencing compared to traditional thalassemia gene testing from 100 cases with clinical evidence of suspected thalassemia. The variants including SNVs, rare deletions and triplications in the *HBA1* and *HBA2* genes. The results further validated the third-generation sequencing utilized in this study as a promising genetic testing method for thalassemia carrier diagnosis, especially for rare variants identification.

## Supplementary Information

Supplementary Information 1.

Supplementary Information 2.

Supplementary Information 3.

## Data Availability

The datasets generated during the current study are available in the SRA database of National Center for Biotechnology Information (Accession: PRJNA836009, ID: 836009) (https://www.ncbi.nlm.nih.gov/sra/PRJNA836009).
