# Supplementary figures and images for: Analysis of rare thalassemia genetic variants based on third-generation sequencing
Source: Sci Rep. 2022 Jun 14;12:9907. doi: 10.1038/s41598-022-14038-8 (PMC9197973; doi:10.1038/s41598-022-14038-8)

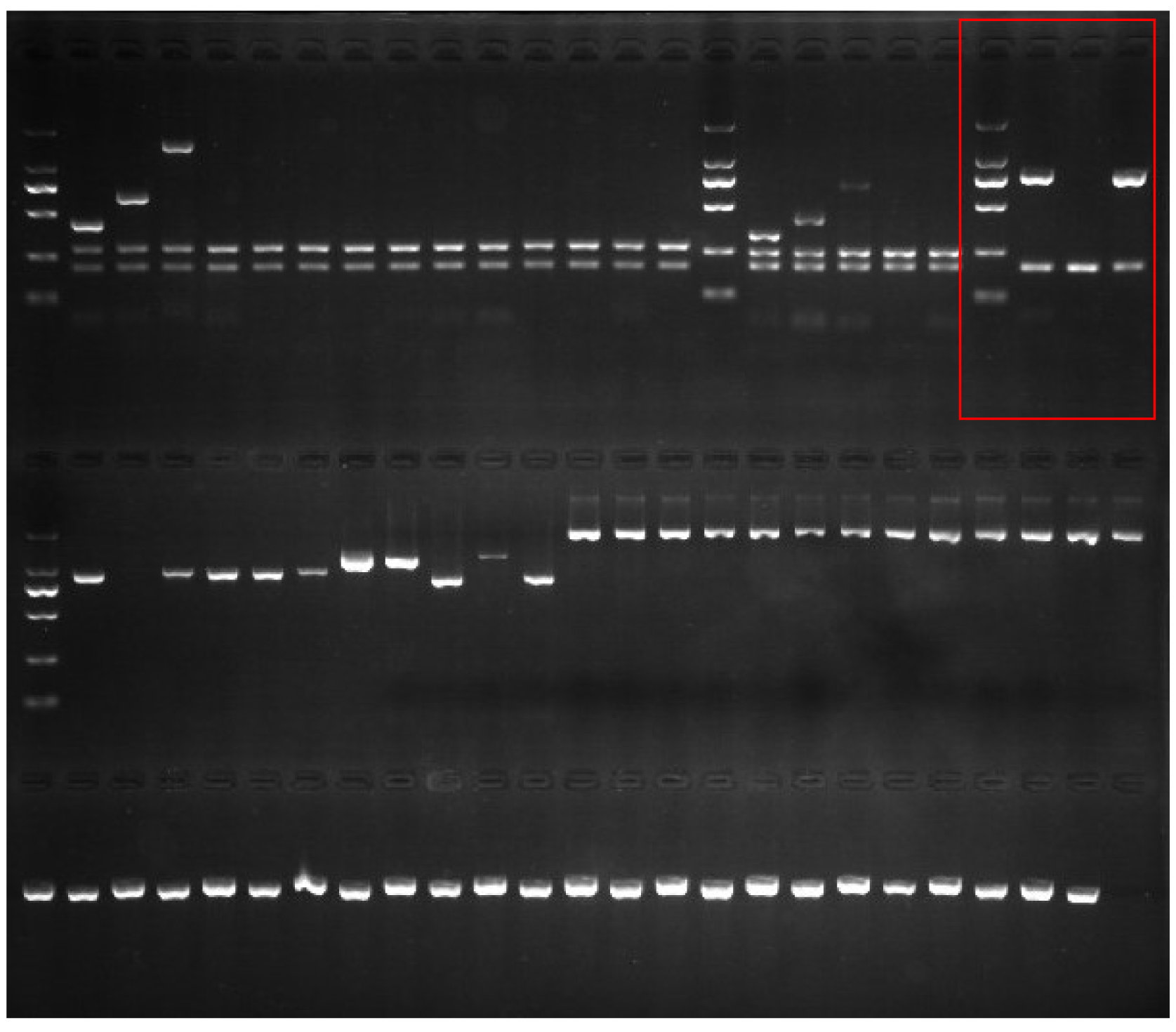

Supplement: Supplementary file 1 — Supplementary Information 1. [file 41598_2022_14038_MOESM1_ESM.tif]

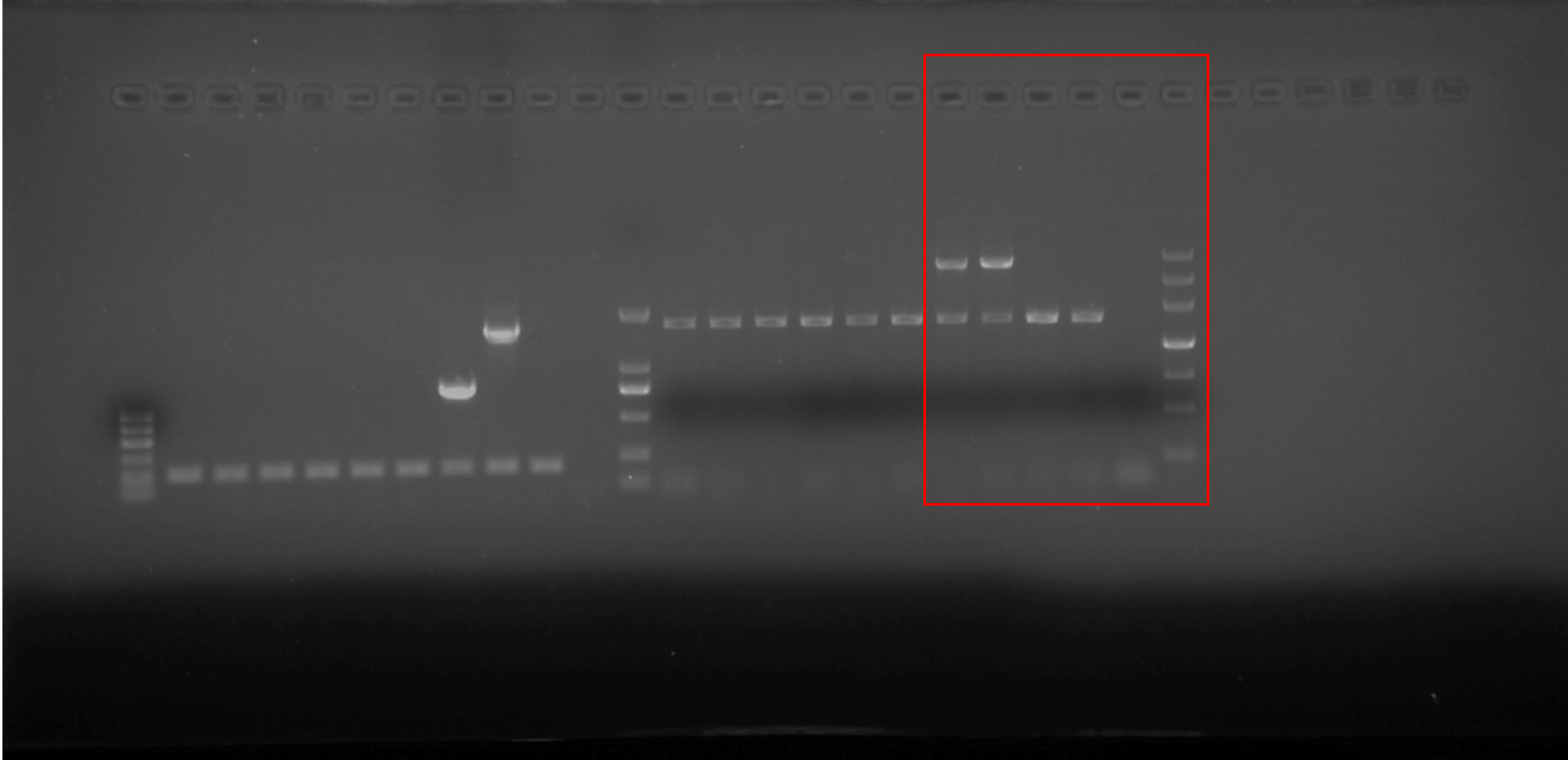

Supplement: Supplementary file 2 — Supplementary Information 2. [file 41598_2022_14038_MOESM2_ESM.tif]
