# Supplementary material for: Analysis of rare thalassemia genetic variants based on third-generation sequencing
Source: Sci Rep. 2022 Jun 14;12:9907. doi: 10.1038/s41598-022-14038-8 (PMC9197973; doi:10.1038/s41598-022-14038-8)
Supplement: Supplementary file 3 — Supplementary Information 3. [file 41598_2022_14038_MOESM3_ESM.docx]

Analysis of rare thalassemia genetic variants based on third-generation sequencing

Cuiting Peng^1,2^, Haixia Zhang^1,2^, Jun Ren^1,2^, Han Chen^1,2^, Ze Du^1,2^, Tong Zhao^1,2^, Aiping Mao^3^, Ruofan Xu^3^, Yulin Lu^3^, He Wang^1,2^, Xinlian Chen^1,2,*^, Shanling Liu^1,2,*^

**Supplementary Figure 1. The original gels for identification of multiple mutations in the polyadenylation signal site and compound --^SEA^/αα.**

The cropped gel of Figure 2C was framed in red line.

**Supplementary Figure 2. The original gels for verification of α-globin gene triplication.**

The cropped gel of Figure 5B was framed in red line.
